# Drug-Coated Balloon Treatment After Lesion Preparation in Selected Acute Coronary Syndrome Lesions: A Dual-Center Real-World Registry

**DOI:** 10.3390/jcdd13080375

**Published:** 2026-08-07

**Authors:** Ivana Jurin, Hrvoje Jurin, Joško Bulum, Zoya Jelovečki Đokić, Irzal Hadžibegović, Šime Manola, Tomislav Krčmar, Denis Došen, Kristina Marić Bešić

**Affiliations:** 1 Department of Cardiology, University Hospital Dubrava, Avenija Gojka Šuška 6, 10000 Zagreb, Croatia; irzalh@gmail.com (I.H.);; 2 Department of Cardiology, University Hospital Centre Zagreb, Kišpatićeva 12, 10000 Zagreb, Croatiajbulum@gmail.com (J.B.);; 3 School of Medicine, University of Zagreb, Šalata 3, 10000 Zagreb, Croatia; 4 University Hospital Centre Sestre Milosrdnice, Vinogradska Cesta 29, 10000 Zagreb, Croatia; 5 Faculty of Dental Medicine and Health, Josip Juraj Strossmayer University of Osijek, Crkvena 21, 31000 Osijek, Croatia; 6 Faculty of Medicine, University of Rijeka, Braće Branchetta 20, 51000 Rijeka, Croatia

**Keywords:** acute coronary syndrome, drug-coated balloon, lesion preparation, percutaneous coronary intervention, real-world registry, target lesion revascularization

## Abstract

Drug-coated balloon (DCB) PCI can avoid permanent metallic scaffolding in selected acute coronary syndrome (ACS) lesions, but interpretation depends on lesion preparation, procedural selection, and follow-up ascertainment. We analyzed a retrospective dual-center registry of 276 unique patients who underwent one index DCB-treated ACS procedure after operator-judged adequate lesion preparation. Presentations were NSTEMI in 174 patients (63.0%), STEMI in 90 (32.6%), and unstable angina in 12 (4.3%). Paclitaxel-coated DCBs were used in 262 patients (94.9%) and sirolimus/non-paclitaxel DCBs in 14 (5.1%). Thrombus aspiration and GP IIb/IIIa inhibitors were used selectively in 17 (6.2%) and 28 (10.1%) patients, respectively. Bail-out stenting was required in 13 patients (4.7%). Predominantly benign/non-flow-limiting angiographic dissection was recorded in 18 patients (6.5%). A follow-up of at least 365 days or death within 365 days was available for 274 patients (99.3%). Clinically driven TLR occurred in 18 patients (crude 18/274, 6.6%; Kaplan–Meier 365-day estimate, 6.8%; 95% CI, 4.3–10.5), all occurring between 3 and 9 months. All-cause death occurred in 12 patients (Kaplan–Meier estimate, 4.4%; 95% CI, 2.5–7.5). In selected ACS lesions that passed a preparation gate, DCB treatment was associated with feasible procedural results and acceptable 12-month clinically recorded outcomes. The findings do not estimate all-comer DCB eligibility and should not be interpreted in terms of the efficacy of DCBs compared with DESs.

## 1. Introduction

Drug-eluting stents (DESs) remain the standard device platform for PCI in acute coronary syndrome (ACS), because they provide predictable acute luminal gain and durable antirestenotic efficacy [1]. However, a permanent metallic scaffold can also be associated with late device-related events, and there is increasing interest in selected stent-free strategies when the acute angiographic result is already acceptable [2].

Drug-coated balloons (DCBs) deliver an antiproliferative drug without leaving a permanent implant. Their established role in in-stent restenosis has expanded toward selected de novo lesions, particularly when adequate lesion preparation, preserved flow, limited recoil, and no flow-limiting dissection can be achieved [3,4]. Randomized and observational DCB evidence in ACS remains conditional on lesion suitability rather than diagnostic category alone, as shown in selected small-vessel, STEMI, NSTEMI, and registry cohorts [5,6,7,8,9,10,11,12].

The central uncertainty in ACS is therefore not whether all culprit lesions should be treated without a stent but whether a lesion that has passed a procedural preparation gate can be accepted for a DCB-only or DCB-first result. This distinction is especially important in retrospective registries, where the denominator often begins after treatment selection rather than at all-comer ACS admission.

We analyzed a retrospective, dual-center registry of operator-selected ACS culprit or clinically relevant target lesions treated with DCBs after lesion preparation. The aim was descriptive and procedural: to report preparation criteria, angiographic results, device coating, adjunctive thrombus management, bail-out stenting, and 12-month time-to-event outcomes in preparation-qualified lesions. This study was not designed to estimate all-comer DCB eligibility or to compare DCBs with DESs.

## 2. Materials and Methods

### 2.1. Study Design and Population

This retrospective observational analysis used a pooled dual-center registry from two tertiary cardiac centers. The cohort comprised 276 unique adult patients with STEMI, NSTEMI, or unstable angina who underwent one index DCB-treated ACS procedure between November 2021 and December 2024. Each patient contributed only one index procedure to the analysis. If more than one lesion was treated during the index ACS episode, the clinically relevant DCB-treated target lesion/procedure was retained as the index entry.

The registry denominator began at DCB delivery. Therefore, it did not capture all ACS admissions, all lesions considered for a DCB-first strategy, lesions rejected before DCB delivery, or lesions converted to another device strategy before DCB use. Consequently, bail-out stenting after DCB delivery was retained in the analytic cohort, whereas pre-DCB treatment failures or rejected lesions were not part of the denominator.

**Figure 1:**
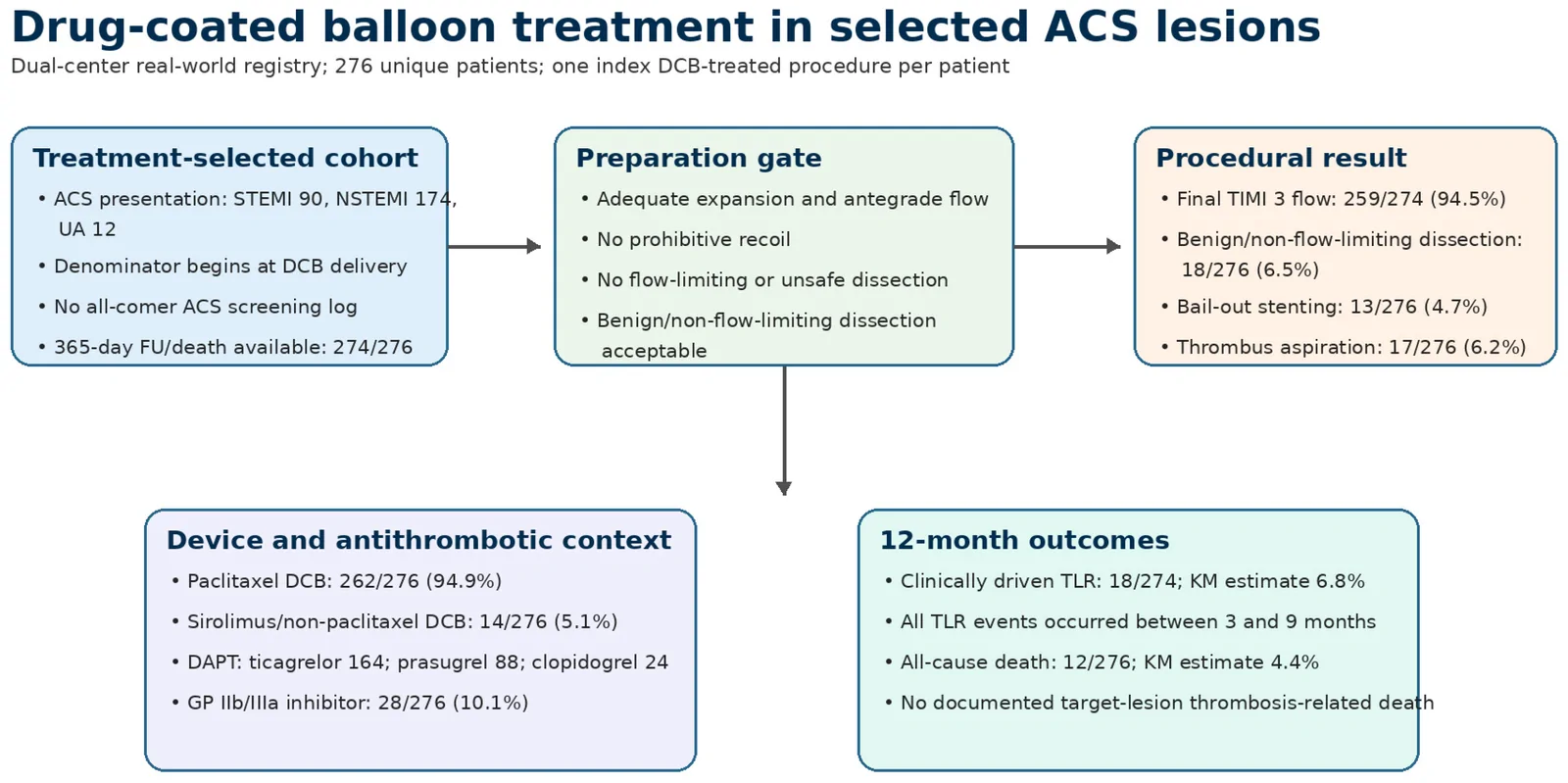
Central illustration. Retrospective study population, preparation gate, procedural benchmarks, device and antithrombotic context, and 12-month outcomes. Procedural dissection and target lesion revascularization are displayed in separate panels because they are conceptually distinct despite having the same absolute count. ACS, acute coronary syndrome; DAPT, dual antiplatelet therapy; DCB, drug-coated balloon; FU, follow-up; GP, glycoprotein; PCI, percutaneous coronary intervention; TIMI, thrombolysis in myocardial infarction; TLR, target lesion revascularization. Arrows indicate the sequence from the treatment-selected cohort through the preparation gate to procedural and clinical outcome domains.

A clinically relevant target lesion was defined as an ACS-related lesion treated with DCBs during the index episode because it was considered responsible for the presentation, contributory to ischemia, or requiring clinically indicated treatment in the same procedural strategy. This included culprit lesions and selected non-culprit but clinically relevant lesions treated in the same ACS treatment pathway; the analysis was not intended to compare culprit versus non-culprit lesion outcomes. The study framework is summarized in Figure 1.

### 2.2. Lesion Preparation and DCB Strategy

The treating interventional cardiologist decided whether to proceed to DCB delivery after angiography and lesion preparation. Suitability required visually manageable thrombus, restored or maintained antegrade flow, acceptable lesion expansion, no prohibitive acute recoil, and no flow-limiting or otherwise unsafe dissection by operator assessment. Benign/non-flow-limiting dissections were accepted when antegrade flow was preserved and the result was judged stable.

Preparation was conducted stepwise with semi-compliant and/or non-compliant balloons. Scoring or cutting balloons were used selectively for resistant, fibrotic, or calcified lesions. DCB diameter and length were selected by the operator according to angiographic vessel size and lesion length. Bail-out stenting was performed for an unacceptable acute result, including flow-limiting dissection, unsafe recoil, thrombotic complication, or inability to accept a stent-free final result.

### 2.3. Antithrombotic Treatment and Thrombus Management

Intraprocedural anticoagulation followed local ACS PCI protocols and was based on unfractionated heparin. GP IIb/IIIa inhibitors and thrombus aspiration were not routine procedural adjuncts; they were used selectively at the operator’s discretion in the setting of high thrombus burden, slow/no reflow, or thrombotic complications.

At discharge, dual antiplatelet therapy (DAPT) consisted of aspirin plus ticagrelor, prasugrel, or clopidogrel. Potent P2Y12 inhibition with ticagrelor or prasugrel was preferred unless contraindicated, not tolerated, or considered clinically unsuitable because of bleeding risk, age, concomitant oral anticoagulation, or other clinical considerations. Intended DAPT duration followed contemporary ACS practice and individual risk assessment.

### 2.4. Angiographic Variables and Definitions

Clinical, angiographic, procedural, and follow-up variables were extracted from the source registry and electronic health records. Recorded variables included coronary territory, target segment, visual stenosis category, pre- and postprocedure TIMI flow, number and dimensions of DCBs, use of scoring/cutting balloons, intravascular imaging, residual stenosis category, angiographic dissection, bail-out stenting, and clinical follow-up.

Functional complete occlusion was defined angiographically by preprocedure TIMI grade 0 flow, irrespective of whether a morphologically complete 100% stenosis was visually present. Visual 100% stenosis was analyzed separately as a morphological angiographic estimate. This distinction was used because thrombus, subtotal stenosis, vasospasm, or transient flow impairment in ACS may produce functional occlusion without a fixed complete anatomical obstruction [13].

Dissection was recorded as present or absent and was interpreted pragmatically by the treating operator. Formal NHLBI A-F grading, core laboratory assessment, and QCA measurements were not available. A strict optimal stent-free procedural result was defined as DCB completion without bail-out stenting, final TIMI grade 3 flow, residual stenosis ≤30%, and the absence of flow-limiting or otherwise prohibitive dissection by operator assessment.

### 2.5. Endpoints and Follow-Up

The primary endpoint was clinically driven target lesion revascularization (TLR) within 365 days. TLR was defined as repeat PCI or coronary bypass surgery involving the DCB-treated lesion or adjacent treated segment after recurrent symptoms, objective ischemia, recurrent ACS, or angiographic restenosis judged clinically relevant by the treating cardiologist. Events were identified from hospital and outpatient records and were not adjudicated by an independent clinical event committee.

Repeat angiography was not protocol-mandated. It was performed according to the clinical judgment of the treating cardiologist rather than a prespecified schedule. Where records permitted, the provenance of TLR was categorized as recurrent symptoms/angina, objective ischemia, recurrent ACS, or clinically guided angiographic finding/staged assessment. No mandatory ischemia testing was required before repeat revascularization.

Secondary outcomes were all-cause death, recurrent ACS hospitalization, stroke, major bleeding, and a pragmatic clinical composite defined as the per-patient occurrence of any of the following within 365 days: all-cause death, recurrent ACS, stroke, or major bleeding. No patient had more than one composite component within the 365-day window; therefore, the composite count (15/276) represents 15 unique patients and not a sum of overlapping events. Major bleeding was defined according to the major bleeding events recorded in the source clinical documentation; formal BARC subtype adjudication was not available, and minor or nuisance bleeding was not included. The primary analytic window was 365 days to provide a uniform horizon across patients. Longer observed follow-up was used to verify survival and censoring status, but events beyond 365 days were not analyzed because longer-term event ascertainment was not uniformly structured across centers.

### 2.6. Statistical Analysis

Continuous variables are reported as the mean ± standard deviation or median (interquartile range) and categorical variables as number (percentage). Exact binomial 95% confidence intervals (CIs) were calculated for crude proportions, including zero-event outcomes. Between-group exploratory comparisons used chi-square, Fisher exact, Student t, or Mann–Whitney tests as appropriate; no inference was drawn from sparse subgroup comparisons.

Kaplan–Meier analyses were performed for 365-day clinically driven TLR and all-cause death, with patients censored at the earliest point of last contact or 365 days. Death before TLR was treated as censoring in the Kaplan–Meier TLR analysis and as a competing event in a descriptive cumulative incidence sensitivity analysis. Kaplan–Meier estimates are reported with 95% CIs based on Greenwood standard errors using log–log transformation. Number-at-risk data are shown at baseline and 30, 90, 180, 270, and 365 days and represent patients still under observation and event-free immediately before each landmark; events occurring on a landmark day were counted after that time point for the number-at-risk table. Because event counts were low and treatment selection was operator-directed, preparation device and coating analyses were descriptive and hypothesis-generating. All statistical analyses were performed using Python version 3.13.5 (Python Software Foundation, Wilmington, DE, USA).

### 2.7. Ethical Considerations

This study was conducted in accordance with the Declaration of Helsinki and approved by the Ethics Committee of University Hospital Dubrava, Croatia (approval No. 2020/2409-08; date of approval: 24 September 2020), and by the Ethics Committee of University Hospital Centre Zagreb, Croatia (Class: 8.1-23/302-2; No.: 02/013 AG; date of approval: 4 December 2023). Written informed consent for participation and access to relevant medical records was obtained according to institutional procedures.

## 3. Results

### 3.1. Clinical Characteristics

The cohort included 276 unique patients who each contributed one index DCB-treated ACS procedure. The mean age was 67.2 ± 11.5 years, and 192 patients (69.6%) were men. NSTEMI was the presentation in 174 patients (63.0%), STEMI in 90 (32.6%), and unstable angina in 12 (4.3%) (Table 1).

### 3.2. Angiographic and Procedural Findings

The target territory was the left circumflex/obtuse marginal system in 114 patients (41.3%), left anterior descending/diagonal system in 86 (31.2%), right coronary/posterior descending/posterolateral system in 58 (21.0%), and ramus intermedius in 18 (6.5%). Bifurcation lesions were present in 58 patients (21.0%) (Table 2).

Preprocedure TIMI grade 0 flow was present in 49 patients (17.8%; 95% CI, 13.4–22.8), whereas visual 100% stenosis was recorded in 37 (13.4%). In STEMI, TIMI grade 0 flow was present in 42 of 90 patients (46.7%). Postprocedure TIMI grade 3 flow was achieved in 259 of 274 patients with recorded final flow (94.5%; 95% CI, 91.1–96.9). In two patients, final TIMI flow could not be coded from the structured procedural record and was treated as missing for this variable only; both patients remained included in the clinical cohort and all other outcome analyses. Additional lesion segment and ACS presentation details are provided in Supplementary Tables S1 and S2.

Paclitaxel-coated DCBs were used in 262 patients (94.9%), whereas 14 patients (5.1%) received at least one sirolimus-coated/non-paclitaxel DCB. Scoring or cutting balloons were used in 87 procedures (31.5%), and inflation lasted at least 60 s in 252 (91.3%). IVUS or OCT was used in 33 patients (12.0%). Residual stenosis >30% was recorded in 28 patients (10.1%). Predominantly benign/non-flow-limiting angiographic dissection was recorded in 18 patients (6.5%). Bail-out stenting was required in 13 patients (4.7%).

Thrombus aspiration was performed selectively in 17 patients (6.2%), and GP IIb/IIIa inhibitors were used selectively in 28 (10.1%). At discharge, DAPT consisted of aspirin plus ticagrelor in 164 patients (59.4%), prasugrel in 88 (31.9%), and clopidogrel in 24 (8.7%). Major bleeding was rare (2/276, 0.7%): one event occurred in a patient discharged on ticagrelor and one in a patient discharged on prasugrel, whereas no major bleeding occurred among clopidogrel-treated patients. This P2Y12-stratified safety summary was descriptive only because treatment allocation was not randomized and events were sparse.

### 3.3. Clinical Outcomes and Endpoint Ascertainment

A follow-up of at least 365 days or death within 365 days was available in 274 patients (99.3%). Two patients without complete 365-day follow-up were censored at the last contact in time-to-event analyses. The median observed follow-up was 23.8 months (IQR, 15.1–35.6), but primary endpoint reporting was restricted to the 365-day analytic window (Table 3).

Clinically driven TLR occurred in 18 of 274 evaluable patients (6.6%; exact 95% CI, 3.9–10.2). The 365-day Kaplan–Meier estimate for TLR was 6.8% (95% CI, 4.3–10.5), and the death competing cumulative incidence sensitivity estimate was 6.5%. All TLR events occurred between 3 and 9 months after the index procedure; no TLR occurred within the first 90 days or after month 9 within the 365-day analytic window.

**Figure 2:**
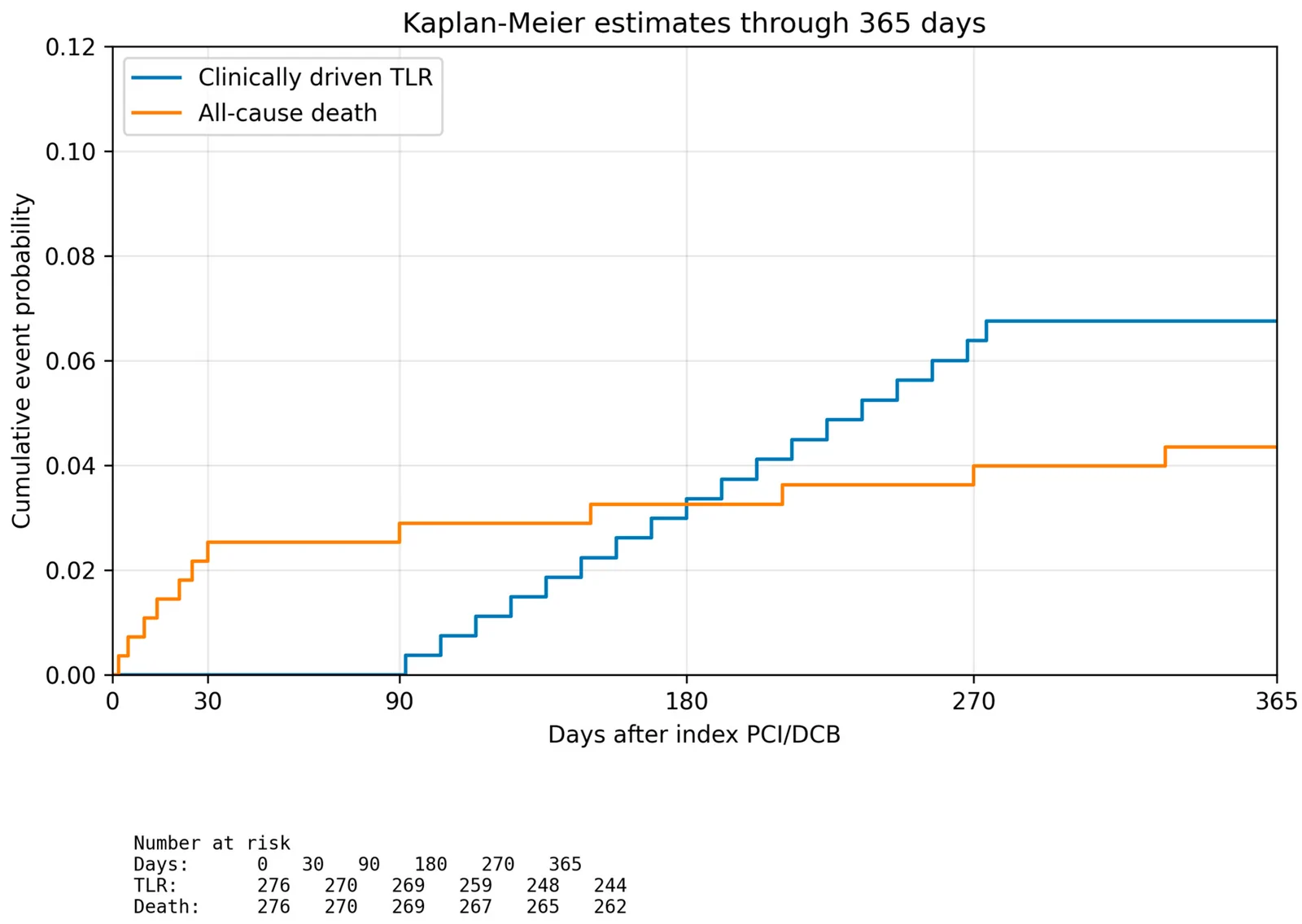
Kaplan–Meier estimates for clinically driven target lesion revascularization and all-cause death through 365 days. Number-at-risk values are shown below the plot and were counted immediately before each landmark day; one death occurred on day 30 and therefore remains included in the day-30 at-risk count before being removed after that time point. Death before target lesion revascularization was handled as censoring in the Kaplan–Meier analysis and as a competing event in the sensitivity cumulative incidence analysis. Events occurring on a landmark day are counted after that time point for the number-at-risk table.

TLR was triggered by recurrent symptoms or angina in 11 patients, objective ischemia in 2, recurrent ACS in 1, and clinically guided angiographic finding or staged assessment in 4. Recurrent ACS was recorded only when a new ACS hospitalization occurred during follow-up; therefore, most TLR events were not recurrent ACS presentations. Repeat angiography was performed in 102 patients (37.0%). All 18 TLR events occurred among patients who underwent repeat angiography (18/102, 17.6%), whereas no TLR was recorded among patients without repeat angiography (0/174). This descriptive stratification should not be interpreted as a causal risk comparison because repeat angiography was clinically driven and often represented the diagnostic pathway leading to TLR. TLR provenance and angiographic follow-up ascertainment are summarized in Supplementary Table S8. The Kaplan–Meier estimates are shown in Figure 2.

All-cause death occurred in 12 patients (4.3%; exact 95% CI, 2.3–7.5), with a 365-day Kaplan–Meier estimate of 4.4% (95% CI, 2.5–7.5). Seven deaths occurred within 30 days, including one event on day 30, and five occurred between days 31 and 365. Death occurred in 7 of 90 STEMI patients (7.8%) and 5 of 186 NSTEMI/unstable angina patients (2.7%). The cause of death was cardiovascular in six patients, non-cardiovascular in four, and undetermined in two. No death was documented as definitely related to thrombosis of the DCB-treated target lesion. Recurrent ACS occurred in one patient (0.4%), stroke in zero, and major bleeding in two (0.7%). Death timing and cause are summarized in Supplementary Table S10.

### 3.4. Exploratory Preparation, Coating, and Bail-Out Analyses

Residual stenosis >30% was less frequent after scoring or cutting balloon preparation than after conventional balloon preparation (2 of 87 [2.3%] vs. 26 of 189 [13.8%]; *p* = 0.002). Because device selection was operator-directed and informed by lesion appearance, this association should be interpreted as hypothesis-generating and not as evidence of a causal treatment effect. An exploratory preparation device comparison is provided in Supplementary Table S3.

Coating-specific outcomes were sparse. Among 262 patients treated with paclitaxel-coated DCBs only, TLR occurred in 17 (6.5%), all-cause death in 12 (4.6%), and bail-out stenting in 12 (4.6%). Among 14 patients with any sirolimus/non-paclitaxel DCB exposure, TLR occurred in 1 (7.1%), all-cause death in 0, and bail-out stenting in 1 (7.1%). No formal comparison was performed. Coating categories and outcomes are summarized in Supplementary Table S9.

Among 263 DCB-only procedures, TLR occurred in 16 (6.1%), all-cause death in 11 (4.2%), and major bleeding in 2 (0.8%). Among the 13 bail-out-stented procedures, TLR occurred in 2 (15.4%), all-cause death in 1 (7.7%), and major bleeding in 0. These subgroup data are descriptive because bail-out stenting was selected by the acute procedural result. Final treatment status subgroups are summarized in Supplementary Table S11.

## 4. Discussion

### 4.1. Principal Findings and Contribution

This registry addresses a practical question that is increasingly encountered in ACS PCI: what outcomes can be expected when an ACS lesion has already passed a preparation gate and is accepted for DCB delivery? In 276 unique patients treated during one index ACS procedure, DCB treatment after lesion preparation was associated with high final TIMI grade 3 flow, infrequent bail-out stenting, predominantly benign/non-flow-limiting dissections, and a 365-day Kaplan–Meier TLR estimate of 6.8%. The results support feasibility in preparation-qualified lesions, but they do not imply that DCBs should replace DESs in unselected ACS or that the proportion of all ACS lesions eligible for DCB treatment can be inferred from this cohort.

The additional contribution of this study is not a new comparison with DESs but a more granular description of the clinical and procedural conditions under which a stent-free result was accepted. First, the denominator is explicitly treatment-selected and begins at DCB delivery, not at all-comer ACS admission. Second, bail-out stenting is retained in the analytic cohort, rather than excluded to make the DCB result appear cleaner. Third, endpoint ascertainment is linked to clinically driven repeat angiography, making clear that TLR represents clinically recorded revascularization rather than systematic angiographic restenosis. Fourth, device coating, thrombus management adjuncts, DAPT, bleeding, and death classification are reported together, allowing the outcomes to be interpreted within a realistic ACS treatment context. A contextual comparison with selected DCB evidence is provided in Supplementary Table S4.

### 4.2. Current Evidence and Position of Present Registry

DES remains the standard device platform for ACS PCI because it provides reliable acute scaffolding and antirestenotic efficacy [1]. The rationale for DCBs is different: they aim to deliver an antiproliferative drug without leaving a permanent implant, thereby avoiding the use of additional metal when the acute result is already acceptable [2,3,4]. This concept is established for in-stent restenosis and selected de novo disease, but ACS remains more conditional because thrombus, plaque disruption, vasomotor tone, and microvascular impairment can make the immediate result less predictable.

The available ACS evidence is therefore best read through the lens of selection. BASKET-SMALL 2 provides randomized small-vessel data, with a prespecified ACS analysis [5,9]. REVELATION studied a highly selected STEMI population in which randomization required adequate predilatation and limited residual stenosis, and bail-out stenting remained part of the procedural reality [6,7]. PEPCAD NSTEMI extended the randomized ACS experience to non-ST-elevation presentations [8]. Observational STEMI and broader ACS registries have further supported feasibility, but their interpretation depends strongly on whether the denominator includes all attempted cases, only uncomplicated cases, or only procedures completed with DCB delivery [10,14,15,16,17]. Against this background, the present registry adds a mixed ACS, dual-center, post-preparation cohort with the explicit reporting of bail-out stenting, follow-up-dependent TLR ascertainment, device coating, and antithrombotic context.

### 4.3. The Preparation Gate in ACS DCB PCI

The central procedural step in DCB PCI is the decision to accept or reject the acute result after lesion preparation. In ACS, this decision is more nuanced than simply identifying the culprit lesion. A functionally occluded vessel by TIMI flow does not always correspond to a fixed morphological 100% stenosis, and thrombus or vasomotor changes can produce transient flow impairment. This distinction is relevant because DCB efficacy depends on sufficient drug transfer to the vessel wall after adequate expansion, while the balloon itself cannot correct persistent recoil or stabilize an unsafe dissection.

In this cohort, final TIMI grade 3 flow was achieved in 94.5%, residual stenosis >30% was recorded in 10.1%, and bail-out stenting was performed in 4.7%. The low bail-out rate should be interpreted within the selected denominator: it reflects lesions that reached DCB delivery after operator-assessed preparation, not the failure rate of an all-comer DCB-first strategy. Similarly, the 6.5% dissection rate refers to dissections accepted as predominantly benign/non-flow-limiting or otherwise managed by the operator, not to a systematically graded dissection burden. Contemporary DCB consensus documents emphasize these procedural variables, including flow, recoil, residual stenosis, dissection, imaging, and bail-out criteria, as essential elements for interpreting DCB studies [18].

### 4.4. TLR, Repeat Angiography, and Clinical Ascertainment

The 365-day time-to-event analysis provides a clearer estimate of 12-month outcome than crude proportions alone. Clinically driven TLR occurred in 18 patients, all occurring between 3 and 9 months, corresponding to a 365-day Kaplan–Meier estimate of 6.8% and a competing risk cumulative incidence of 6.5%. This rate appears plausible for a mixed, real-world ACS cohort and is higher than the very low event rates observed in highly selected randomized STEMI settings while remaining compatible with broader registry experience.

The more important point is how TLR was detected. Repeat angiography was not protocol-mandated and was performed in 37.0% of patients according to clinical judgment. All recorded TLR events occurred among patients who underwent repeat angiography. This finding should not be interpreted as evidence that patients without repeat angiography had no restenosis. Rather, it shows that the endpoint is clinically and angiographically ascertainment-dependent: patients with recurrent symptoms, ischemia, recurrent ACS, or clinical concern were more likely to undergo angiography and therefore more likely to have a lesion identified and treated. For this reason, the endpoint should be understood as clinically recorded TLR in routine practice, not as a systematic restenosis rate.

### 4.5. Device Coating, Antithrombotic Context, and Safety

Most procedures used paclitaxel-coated DCBs, whereas sirolimus-coated/non-paclitaxel exposure was uncommon. Reporting this distinction is important because DCBs are not interchangeable purely by name: drug, excipient, dose density, release kinetics, and transfer technology may all influence performance [4,15,18]. However, only 14 patients received a sirolimus/non-paclitaxel device, so the similar descriptive TLR rates across coating categories cannot be interpreted as comparative evidence. Brand-level and drug dose density analyses were not possible because these fields were not uniformly captured in structured registry data.

The antithrombotic and thrombus management data also make the safety interpretation more clinically coherent. DAPT consisted predominantly of ticagrelor or prasugrel, with clopidogrel used in selected patients in whom potent P2Y12 inhibition was unsuitable. GP IIb/IIIa inhibitors and thrombus aspiration were used selectively rather than routinely, consistent with contemporary ACS practice in which these adjuncts are reserved for high thrombus burden, slow/no reflow, or thrombotic complications. Major bleeding was rare and occurred only in patients receiving potent P2Y12 inhibition, but the event count is too small and treatment selection too confounded to infer drug-specific safety. Antithrombotic and thrombus management details are summarized in Supplementary Table S7.

### 4.6. Lesion Preparation, Scoring/Cutting Balloons, and Imaging

The observed association between scoring/cutting balloon use and lower residual stenosis >30% is biologically plausible but not causal evidence. Operators selected modified balloons based on lesion appearance, resistance, calcification, or recoil, and these features were not captured with enough anatomical granularity to separate treatment effect from indication. This result should therefore be viewed as a hypothesis-generating signal that preparation technique matters, not as proof that scoring or cutting balloons independently improve angiographic results in ACS DCB PCI. Recent DCB-only STEMI data have likewise highlighted the need to treat scoring balloon findings cautiously when the preparation strategy is operator-selected [19].

The limited use of IVUS or OCT is another important constraint. Angiography alone can underestimate vessel size, calcium, plaque morphology, intramural hematoma, and dissection extent. The recent imaging and DCB consensus literature supports more standardized lesion assessment, endpoint definitions, and imaging-guided sizing in future DCB studies [18,20,21,22]. The present registry reflects real-world ACS practice but also shows why future prospective work should integrate systematic lesion morphology, dissection grading, and imaging-based optimization.

### 4.7. Limitations and Interpretation

This study remains retrospective and treatment-selected. The denominator begins at DCB delivery and does not include all ACS admissions, all lesions considered for DCB treatment, lesions rejected before DCB delivery, or lesions converted to another device strategy before DCB use. Therefore, this study cannot estimate all-comer eligibility for DCB treatment or the intention-to-treat failure rate of a DCB-first ACS strategy. Data element limitations and denominator rules are summarized in Supplementary Tables S5 and S6.

Angiographic assessment was operator-based, without a core laboratory, QCA, systematic thrombus or calcium grading or formal NHLBI dissection classification. Repeat angiography was clinically driven and performed in a minority of patients, introducing the follow-up-dependent ascertainment of TLR. Events were not adjudicated by an independent committee, and objective ischemia testing was not mandatory before TLR. Coating-specific, DAPT-stratified bleeding, and bail-out subgroup analyses were descriptive and underpowered. Finally, there was no DES control group, so comparative efficacy or safety cannot be inferred. These limitations define the appropriate interpretation of this study: it is a procedural and clinical benchmark for selected, preparation-qualified ACS lesions, not a comparative effectiveness trial.

## 5. Conclusions

In this treatment-selected dual-center registry of 276 unique ACS patients, DCB treatment after lesion preparation was feasible when the acute result was judged suitable for a stent-free strategy. Bail-out stenting occurred in 4.7%, recorded dissections were predominantly benign/non-flow-limiting, and the 365-day Kaplan–Meier TLR estimate was 6.8%. These findings support the feasibility of a preparation-gated DCB strategy in selected ACS lesions while emphasizing that the data do not define all-comer eligibility and do not establish the comparative efficacy of DCBs versus DES.

## Figures and Tables

**Table 1:** Baseline clinical characteristics.

Variable	Overall (N = 276)
Age, years	67.2 ± 11.5
Body mass index, kg/m^2^	28.6 ± 4.8 (n = 274)
Male sex	192 (69.6)
Diabetes mellitus	88 (31.9)
Hypertension	227 (82.2)
Hyperlipidemia	212 (77.1; n = 275)
Current smoking	106 (38.4)
Chronic kidney disease	28 (10.1)
Prior myocardial infarction	58 (21.0)
Prior percutaneous coronary intervention	75 (27.2)
Prior coronary artery bypass grafting	11 (4.0)
Clinical presentation	
NSTEMI	174 (63.0)
STEMI	90 (32.6)
Unstable angina	12 (4.3)

Values are mean ± standard deviation or n (%), unless otherwise stated. NSTEMI, non-ST-segment elevation myocardial infarction; STEMI, ST-segment elevation myocardial infarction.

**Table 2:** Angiographic, procedural, device, and antithrombotic characteristics.

Variable	Overall (N = 276)
1-vessel/2-vessel/multivessel disease	91 (33.0)/92 (33.3)/93 (33.7)
Target territory: LAD/diagonal	86 (31.2)
Target territory: LCx/obtuse marginal	114 (41.3)
Target territory: RCA/PDA/RPL	58 (21.0)
Target territory: Ramus intermedius	18 (6.5)
Bifurcation lesion	58 (21.0)
Visual 100% diameter stenosis	37 (13.4)
Preprocedure TIMI grade 0 flow	49 (17.8; 95% CI, 13.4–22.8)
Postprocedure TIMI grade 3 flow	259/274 (94.5; 95% CI, 91.1–96.9)
Drug-coated balloons per procedure	1.16 ± 0.41
Mean DCB diameter, mm	2.60 ± 0.44
Mean DCB length, mm	22.5 ± 5.8
Paclitaxel-coated DCB only	262 (94.9)
Any sirolimus/non-paclitaxel DCB	14 (5.1)
Scoring or cutting balloon	87 (31.5)
Inflation ≥ 60 s	252 (91.3)
Any IVUS or OCT	33 (12.0)
Thrombus aspiration	17 (6.2)
GP IIb/IIIa inhibitor	28 (10.1)
DAPT: Aspirin + ticagrelor	164 (59.4)
DAPT: Aspirin + prasugrel	88 (31.9)
DAPT: Aspirin + clopidogrel	24 (8.7)
Residual stenosis >30%	28 (10.1; 95% CI, 6.8–14.3)
Predominantly benign/non-flow-limiting dissection	18 (6.5; 95% CI, 3.9–10.1)
Acute thrombosis	0 (0.0; 95% CI, 0.0–1.3)
Bail-out stent implantation	13 (4.7; 95% CI, 2.5–7.9)
Strict optimal stent-free result	229 (83.0)
Angiographic follow-up performed	102 (37.0)

Values are the mean ± standard deviation or n (%), unless otherwise stated. CI, confidence interval; DAPT, dual antiplatelet therapy; DCB, drug-coated balloon; GP, glycoprotein; IVUS, intravascular ultrasound; LAD, left anterior descending; LCx, left circumflex; OCT, optical coherence tomography; PDA, posterior descending artery; RCA, right coronary artery; RPL, posterolateral branch; TIMI, thrombolysis in myocardial infarction. The denominator for postprocedure TIMI grade 3 flow is 274 because final TIMI flow could not be coded from the structured procedural record in two patients; these patients remained included in the clinical cohort and all other analyses.

**Table 3:** Follow-up and clinical outcomes at 12 months.

Endpoint	Crude Count/Proportion	365-Day Time-to-Event Estimate or Note
Follow-up ≥ 365 days or death within 365 days	274/276 (99.3)	Two patients censored before 365 days
Median observed follow-up, months	23.8 (15.1–35.6)	Primary analytic window restricted to 365 days
Clinically driven TLR	18/274 (6.6; 95% CI, 3.9–10.2)	Kaplan–Meier 6.8% (95% CI, 4.3–10.5); competing risk CIF 6.5%
All-cause death	12/276 (4.3; 95% CI, 2.3–7.5)	Kaplan–Meier 4.4% (95% CI, 2.5–7.5)
Recurrent ACS	1/276 (0.4; 95% CI, 0.0–2.0)	Crude proportion
Stroke	0/276 (0.0; 95% CI, 0.0–1.3)	Crude proportion
Major bleeding	2/276 (0.7; 95% CI, 0.1–2.6)	Crude proportion
Pragmatic clinical composite	15/276 (5.4; 95% CI, 3.1–8.8)	Crude proportion

Values are the median (interquartile range) or n/N (%). ACS, acute coronary syndrome; CI, confidence interval; CIF, cumulative incidence function; TLR, target lesion revascularization. The pragmatic clinical composite is a per-patient count; no patient had more than one component event within 365 days, so the count represents 15 unique patients and not a sum of overlapping events.

## Data Availability

The data underlying this study are not publicly available because they contain sensitive clinical information and are subject to institutional and ethical restrictions. De-identified aggregated data may be available from the corresponding author upon reasonable request and subject to institutional approval.

## Supplementary Materials

The following supporting information can be downloaded at https://www.mdpi.com/article/10.3390/jcdd13080375/s1. Tables S1–S11 and STROBE checklist. Specifically, the supplementary material includes Table S1: Target-lesion coronary segment distribution; Table S2: Exploratory comparison by acute coronary syndrome presentation; Table S3: Exploratory comparison by scoring or cutting balloon use; Table S4: Contextual comparison with selected drug-coated balloon evidence; Table S5: Registry data elements and interpretive implications; Table S6: Denominator and endpoint interpretation rules; Table S7: Antithrombotic therapy, thrombus management, and bleeding; Table S8: TLR provenance and angiographic follow-up; Table S9: Device coating and clinical outcomes; Table S10: Death timing and cause; Table S11: Outcomes according to final treatment status; and the STROBE checklist.
