# Supplementary material for: Drug-Coated Balloon Treatment After Lesion Preparation in Selected Acute Coronary Syndrome Lesions: A Dual-Center Real-World Registry
Source: J Cardiovasc Dev Dis. 2026 Aug 7;13(8):375. doi: 10.3390/jcdd13080375 (PMC13512925; doi:10.3390/jcdd13080375)
Supplement: Supplementary file 1 [file jcdd-13-00375-s001.zip › jcdd-4458766-checklist.pdf]

STROBE Statement Checklist - Observational Cohort/Registry Study

Manuscript ID: jcdd-4458766

STROBE item	No.	Location	Explanation
Title and abstract	1	Title/Abstract	Title identifies the design as a registry; abstract describes retrospective dual-center design and key outcomes.
Background/rationale	2	Introduction	Rationale for DCB treatment after lesion preparation in selected ACS lesions.
Objectives	3	Introduction	Objective stated as descriptive procedural and 12-month outcome analysis.
Study design	4	Methods 2.1	Retrospective observational dual-center registry.
Setting	5	Methods 2.1	Two tertiary cardiac centers; November 2021 to December 2024.
Participants	6	Methods 2.1	276 unique patients; one index DCB-treated ACS procedure.
Variables	7	Methods 2.3-2.5	Clinical, angiographic, procedural, treatment, follow-up, and endpoint variables defined.
Data sources/measurement	8	Methods 2.4-2.5	Registry and electronic health records; operator-based angiographic assessment.
Bias	9	Methods/Limitations	Treatment-selected denominator, clinical angiographic follow-up, lack of core lab and independent adjudication discussed.
Study size	10	Methods/Results	All eligible registry entries meeting inclusion criteria; N=276.
Quantitative variables	11	Methods/Statistical Analysis	Continuous/categorical summaries and exact CIs; KM analyses.
Statistical methods	12	Methods 2.6	Exact binomial CIs, KM, Greenwood CI, competing-risk sensitivity, exploratory comparisons.
Participants/results	13	Results/Table 1	Baseline cohort described; follow-up denominator stated.
Descriptive data	14	Results/Table 1-2	Clinical and procedural data reported.
Outcome data	15	Results/Table 3/Figure 2	TLR, death, safety outcomes reported.
Main results	16	Results/Discussion	Crude and time-to-event estimates provided.
Other analyses	17	Supplement S8-S11	Angiographic follow-up, coating, bail-out, and preparation analyses.
Key results	18	Discussion	Main feasibility message and limitations.
Limitations	19	Discussion 4.6	Retrospective, selected denominator, angiographic

			ascertainment, no DES comparator.
Interpretation	20	Discussion/Conclusion	Feasibility in preparation-qualified lesions; no comparative claims.
Generalisability	21	Discussion/Limitations	Cannot infer all-comer DCB eligibility.
Funding	22	Back matter	No external funding.
