# Supplementary material for: Drug-Coated Balloon Treatment After Lesion Preparation in Selected Acute Coronary Syndrome Lesions: A Dual-Center Real-World Registry
Source: J Cardiovasc Dev Dis. 2026 Aug 7;13(8):375. doi: 10.3390/jcdd13080375 (PMC13512925; doi:10.3390/jcdd13080375)
Supplement: Supplementary file 1 [file jcdd-13-00375-s001.zip › jcdd-4458766-supplementary-revised.pdf]

Supplementary Materials

Drug-Coated Balloon Treatment after Lesion Preparation in Selected Acute Coronary Syndrome Lesions: A Dual-Center Real-World Registry

Supplementary Table S1. Target-lesion coronary segment distribution

Target segment	Territory	Overall (N=276)	NSTEMI/UA (n=186)	STEMI (n=90)
First obtuse marginal branch	LCx/OM	51 (18.5)	28 (15.1)	23 (25.6)
Mid left anterior descending artery	LAD/diagonal	24 (8.7)	15 (8.1)	9 (10.0)
Proximal left circumflex artery	LCx/OM	23 (8.3)	13 (7.0)	10 (11.1)
First diagonal branch	LAD/diagonal	22 (8.0)	12 (6.5)	10 (11.1)
Mid right coronary artery	RCA/PDA/RPL	22 (8.0)	16 (8.6)	6 (6.7)
Mid left circumflex artery	LCx/OM	21 (7.6)	15 (8.1)	6 (6.7)
Ramus intermedius	Ramus intermedius	18 (6.5)	14 (7.5)	4 (4.4)
Proximal left anterior descending artery	LAD/diagonal	16 (5.8)	13 (7.0)	3 (3.3)
Posterior descending artery	RCA/PDA/RPL	14 (5.1)	10 (5.4)	4 (4.4)
Other segments	Mixed	85 (30.8)	50 (26.9)	35 (38.9)

Values are n (%).

Supplementary Table S2. Exploratory comparison by ACS presentation

Variable	NSTEMI/UA (n=186)	STEMI (n=90)	P value
Age, years	68.3 ± 11.3	64.8 ± 11.6	0.018
Male sex	127 (68.3)	65 (72.2)	0.577
Prior MI	44 (23.7)	14 (15.6)	0.156
Prior PCI	58 (31.2)	17 (18.9)	0.032
Multivessel disease	71 (38.2)	22 (24.4)	0.029
Visual 100% stenosis	5 (2.7)	32 (35.6)	<0.001
Preprocedure TIMI 0 flow	7 (3.8)	42 (46.7)	<0.001
Postprocedure TIMI 3 flow	177 (95.2)	82 (91.1)	0.193
Bail-out stenting	8 (4.3)	5 (5.6)	0.760
Predominantly benign/non-flow-limiting dissection	10 (5.4)	8 (8.9)	0.293
12-month TLR	11 (5.9)	7 (7.8)	0.604
12-month death	5 (2.7)	7 (7.8)	0.063

Exploratory unadjusted comparisons. No center-stratified outcome analysis was performed.

Supplementary Table S3. Exploratory comparison by scoring/cutting balloon use

Variable	Scoring/cutting balloon (n=87)	No scoring/cutting balloon (n=189)	P value
STEMI presentation	23 (26.4)	67 (35.4)	0.167
Preprocedure TIMI 0 flow	10 (11.5)	39 (20.6)	0.089
Visual 100% stenosis	9 (10.3)	28 (14.8)	0.348
Final TIMI 3 flow	83 (95.4)	176 (93.1)	0.595
Residual stenosis >30%	2 (2.3)	26 (13.8)	0.002
Angiographic dissection	8 (9.2)	10 (5.3)	0.293

Variable	Scoring/cutting balloon (n=87)	No scoring/cutting balloon (n=189)	P value
Bail-out stenting	4 (4.6)	9 (4.8)	1.000
12-month TLR	5 (5.7)	13 (6.9)	0.800
12-month pragmatic clinical composite	5 (5.7)	10 (5.3)	1.000

Device selection was operator-directed. The comparison is hypothesis-generating only.

Supplementary Table S4. Contextual comparison with selected DCB evidence

Study	Design	Selection/preparation	Relevance
REVELATION	Randomized selected STEMI trial	Mandatory preparation; bail-out if unsuitable	Selected STEMI feasibility and follow-up
PEPCAD NSTEMI	Randomized NSTEMI trial	DCB after successful preparation	Direct NSTEMI comparative evidence
BASKET-SMALL 2 ACS analysis	Randomized small-vessel trial	Successful predilatation required	Supports selected small-vessel ACS treatment
Merinopoulos STEMI cohort	Real-world DCB-only primary PCI	Selected DCB-only cases	Long-term STEMI feasibility
EASTBOURNE/PEACE	Sirolimus-coated balloon registry	Operator-selected preparation	Acute/chronic syndrome context
Japanese nationwide ACS registry	Propensity-matched registry	Successful/uncomplicated DCB; bail-out excluded	Highlights selection and denominator differences
European multicenter STEMI registry	Real-world STEMI registry	Selected culprit lesions	Multicenter STEMI feasibility

Supplementary Table S5. Data elements that determine interpretation of this registry

Domain	Available in current dataset	Not available / not standardized	Effect on interpretation
Screening denominator	DCB-treated ACS cases (N=276)	All ACS admissions; lesions rejected before DCB delivery	Cannot estimate all-comer eligibility
Lesion morphology	Territory, segment, bifurcation, TIMI flow, visual 100% stenosis	QCA lesion length/RVD, calcification grade, thrombus grade, SYNTAX score	Limits anatomical risk adjustment
Dissection	Present/absent; pragmatic operator assessment	NHLBI grade and core-lab morphology	Limits granular interpretation
Angiographic follow-up	Repeat angiography recorded in 102 patients	No protocol schedule; triggers not prospectively standardized	TLR ascertainment is follow-up-dependent
Clinical endpoints	Clinically recorded TLR and events	Independent event committee, mandatory ischemia testing	Endpoint reflects clinical practice, not adjudicated ischemia-driven TLR
Device coating	Coating category (paclitaxel vs sirolimus/non-paclitaxel)	Brand-level and drug-dose-density fields not uniformly captured	Limits coating-specific interpretation

Supplementary Table S6. Unit of analysis and denominator rules

Item	Value / rule
Total analytic cohort	276 unique patients
Index entry	One index DCB-treated ACS procedure per patient
Primary 365-day endpoint evaluability	274/276 with >=365 days follow-up or death within 365 days
Patients censored before 365 days	2
Center-stratified outcomes	Not performed; pooled dual-center registry
Bail-out stenting	Retained in denominator after DCB delivery

Supplementary Table S7. Antithrombotic and thrombus-management strategy

Variable	Overall (N=276)	Comment
Intraprocedural anticoagulation	Unfractionated heparin-based local ACS PCI protocol	Bivalirudin not used routinely
Thrombus aspiration	17 (6.2)	Selective; not routine
GP IIb/IIIa inhibitor	28 (10.1)	Selective/bail-out use
Aspirin + ticagrelor at discharge	164 (59.4)	Potent P2Y12 inhibitor
Aspirin + prasugrel at discharge	88 (31.9)	Potent P2Y12 inhibitor
Aspirin + clopidogrel at discharge	24 (8.7)	Used when potent P2Y12 inhibition clinically unsuitable
Major bleeding during 365-day follow-up	2 (0.7)	Clinically recorded major bleeding; minor/nuisance bleeding not included
Major bleeding: aspirin + ticagrelor	1/164 (0.6)	Descriptive; no formal comparison
Major bleeding: aspirin + prasugrel	1/88 (1.1)	Descriptive; no formal comparison
Major bleeding: aspirin + clopidogrel	0/24 (0.0)	Descriptive; clopidogrel subgroup small and selected

Supplementary Table S8. Endpoint ascertainment and TLR provenance

Endpoint ascertainment item	n/N (%) or n
Angiographic follow-up performed	102/276 (37.0)
No angiographic follow-up recorded	174/276 (63.0)
TLR among patients with angiographic follow-up	18/102 (17.6)
TLR among patients without angiographic follow-up	0/174 (0.0)
TLR triggered by recurrent symptoms/angina	11/18
TLR triggered by objective ischemia	2/18
TLR triggered by recurrent ACS	1/18
TLR after clinically guided angiographic finding/staged assessment	4/18
TLR solely by routine protocol surveillance	0/18

This table is descriptive. Repeat angiography was clinically driven and may be part of the diagnostic pathway leading to TLR; therefore, it should not be interpreted as a risk comparison between angiography and no-angiography groups.

Supplementary Table S9. Device coating and descriptive 12-month outcomes

Coating category	n	TLR	All-cause death	Bail-out stenting
Paclitaxel-coated DCB only	262	17 (6.5)	12 (4.6)	12 (4.6)
Any sirolimus/non-paclitaxel DCB	14	1 (7.1)	0 (0.0)	1 (7.1)

Brand-level and drug-dose-density analyses were not performed because these variables were not captured uniformly in structured registry data.

Formal hypothesis testing was not performed because non-paclitaxel exposure was infrequent.

Supplementary Table S10. Timing and cause of death within 365 days

Death characteristic	n
All-cause death within 365 days	12
Death in STEMI patients	7/90 (7.8)
Death in NSTEMI/UA patients	5/186 (2.7)
Timing: 0-30 days	7
Timing: 31-365 days	5
Cardiovascular death	6

Death characteristic	n
Non-cardiovascular death	4
Undetermined cause	2
Definitely related to thrombosis of DCB-treated target lesion	0

Supplementary Table S11. Descriptive outcomes by final treatment status

Final treatment status	n	TLR	All-cause death	Major bleeding
DCB-only	263	16 (6.1)	11 (4.2)	2 (0.8)
Bail-out stenting	13	2 (15.4)	1 (7.7)	0 (0.0)

No formal statistical comparison was performed because the bail-out group was small and selected by the acute procedural result.
